# Moral Entrepreneurialism for the Hamburger: Strategies for Marketing a Contested Fast Food

**DOI:** 10.1177/17499755211039932

**Published:** 2021-09-14

**Authors:** Natália Otto, Josée Johnston, Shyon Baumann

**Affiliations:** University of Toronto, Canada

**Keywords:** beef, discourse analysis, ethical consumption, marketing, moral entrepreneurs

## Abstract

Recent research has extended the concept of moral entrepreneurialism to corporate actors. We build on this research to investigate how corporations succeed in this effort by uncovering the strategies and tools they employ as moral entrepreneurs. To do so, we examine the corporate discourse of three prominent fast-food firms to identify how they present hamburgers as good food, in a context where beef is increasingly criticized as morally suspect. Based on a discourse analysis of corporate communications and marketing campaigns, we identify three distinct discursive strategies for managing meat criticisms: (1) global managerialism (McDonald’s); (2) aestheticized simplicity (A&W); and (3) nostalgic, personalized appeals (Wendy’s). These strategies are realized through the use of informational tools to shape what customers think and know about beef, and affective tools to influence how customers feel about beef. Together, these corporate strategies speak to the skilful ability of corporate actors to respond to socio-environmental criticisms. Our case shows how fast-food market actors are able to incorporate critique and offer messages that seek to allow people to feel good about eating beef. This case is relevant to understanding the tools that corporations use to be effective moral entrepreneurs. It also provides a deeper understanding of marketing discourse at the nexus of social problems and consumption choices.

## Introduction

The burger is not only immensely popular, it is also a powerful and contentious symbol of American foodways. While hamburgers can mean different things to different people, they have increasingly come to symbolize an American diet that is meat-intensive, resource-depleting, and unhealthy for humans. They are commonly eaten, but widely invoked as a symbol of the industrial food system’s troubling health and environmental externalities. The burger is prominently featured – visually and textually – in news stories about the environmental costs of eating meat. Consider a *Mother Jones* magazine headline ‘Americans eat six hamburgers a day, and it’s making all of us sicker’ (Oatman, 2017), or *Scientific American*’s use of the term ‘Greenhouse Hamburger’ linking burgers – and meat consumption more broadly – to climate change (Fiala, 2009). In 2014, celebrated food writer Mark Bittman attempted to calculate the true costs of a fast-food hamburger in an article in the *New York Times*, and pinpointed externalities like greenhouse gas emissions, loss of biodiversity, deforestation, water contamination from nitrates and e-coli, as well as numerous health problems. His calculations and investigations led him to conclude the external costs of the burger greatly outweigh the cheap price-tag at the cashier, and he suggests that if producers were forced to take on these costs, ‘the industry would be a highly unprofitable, even silly one. It would either cease to exist or be forced to raise its prices significantly’ (Bittman, 2014). Bittman bluntly concludes that cheeseburgers are the ‘coal of the food world’. Together, these discourses suggest that when you eat a hamburger, you consume a paradoxical food – a meal that is widely appreciated and understood as delicious, but broadly criticized on health and environmental grounds, especially in its fast-food form.

While research has investigated consumers’ conflicted ideas about eating meat (e.g. Oleschuk et al., 2019; Rothgerber, 2018), less is known about how *producers* respond to the ethical challenges facing meat-eaters, and hamburgers in particular. In studying fast-food firms’ messages about hamburgers, our analysis builds on literature on ‘moral entrepreneurialism’ (Becker, 1963), which focuses on efforts to define categories of moral worth and the promotion of moral evaluations. We ask: How do fast-food corporations strategically present the moral worth and aesthetic appeal of burgers in the face of public criticism?

To address this question, we examine the corporate discourse of three prominent fast-food burger chains: McDonald’s (the global burger hegemon), Wendy’s (a mid-sized transnational corporate chain, and A&W (a relatively small chain^1^ in Canada). We use a discourse analysis of corporate communication materials to empirically investigate the discursive strategy each firm employs to present their hamburgers as desirable and as *good* – as a delicious and morally acceptable meal choice. These discursive strategies speak to the skilful ability of corporate actors to respond to socio-environmental criticisms of their products, and reassure consumers who may have ambivalent feelings about eating meat. In documenting these strategies, we show how each strategy relies on a mix of *informational* and *aesthetic* tools to reassure consumers about the moral and aesthetic appeal of hamburgers. Each company also manages what we call a *proliferation/obfuscation tension*: they provide ample information about their meat products to curious consumers, while strategically highlighting simple solutions, obfuscating some of the most serious problems in the food system.

The article proceeds as follows. First, we situate our study in literature concerned with the moral goodness of food and with marketing discourse. We then review key concepts from the moral economy perspective and from literature on moral entrepreneurialism that we rely on. Next, we outline our methods, which primarily employ a discourse analysis of corporate promotions materials in the three cases, supplemented with in-store participant observation. The bulk of the article outlines the three firms’ key strategies for defining burgers as both morally and aesthetically good, and the tools they employ to pursue these strategies. We conclude by summarizing the contribution of our study for understanding the nature of marketing discourse and how it can practise moral entrepreneurialism.

## Marketing A Problematic Food

We approach the study of fast-food marketing within past work that sees media discourses as both reflecting and contributing to culture (Fairclough, 1992; Hall, 1980). Our approach also draws on past findings that marketing relies on an interplay of affect and cognition involving making implicit associations and framing information (Messaris, 1997; Vakratsas and Ambler, 1999). We also work from the understanding that, above all else, marketing seeks to shape consumers’ perceptions and behaviours – a goal that is challenging and omnipresent. Our case study of the fast-food hamburger represents a marketing challenge that has become especially difficult in recent years.

It is useful to briefly outline some of the key challenges to fast-food burger consumption in order to better appreciate how the legitimacy and moral worth of the fast-food burger have been thrown into question. A major public challenge to fast-food burger consumption has centred on its health impacts. Eating meat, especially processed red meats, has been linked to increased health risks like cancer and heart disease (IARC, 2015). Journalists, food scholars, and public health critics have documented the problematic impact of fast-food consumption and a diet high in salt, sugar and fat (e.g. Jaworowska et al., 2013; Moss, 2013; Spurlock, 2006; Winson, 2013). Besides health, meat and meat-production have been criticized for the industrialized production method known as confined animal feeding operations (CAFOs). Industrialized practices allow meat to be produced at a large volume for a low price-tag, effectively making possible the cheap fast-food hamburger. However, academics and journalists alike have linked CAFOs to pressing issues like water contamination, greenhouse gas emissions, antibiotic usage, and poor conditions for animal welfare (e.g. Herrero et al., 2016; Pollan, 2002). As a result, many consumers articulate negative associations with industrial livestock production, even while they continue to eat meat.

In the light of these health, environmental and animal welfare challenges to meat production and consumption, what is known about how the meat industry has responded to these challenges? There is little existing scholarship that looks at producers’ efforts to morally reposition meat production and consumption. One notable exception is Cairns et al. (2015). This research analyses a campaign by the Manitoba Pork Council designed to legitimate pork production and symbolically connect pork to wholesome images of white, heteronormative producer families. Drawing on Sara Ahmed’s concept of ‘affective conversion’, the authors argue that the campaign works at an affective level to reconfigure ‘public feeling toward the hog farming industry—from the shame of harmful and unsafe farming practices to the pride of the (white, heterosexual) family farm’ (Cairns et al., 2015: 1192–1193).

Some food and media scholars have examined the efforts of fast-food firms to augment their social reputation and consumer appeal in the light of social, health and environmental criticisms. For example, Hong (2012) looks at McDonald’s efforts in their corporate social responsibility report to challenge and resist some of the negative associations of the brand; for example, the term ‘McJob’ to describe a dead-end job, and McDonald’s hamburgers have become associated in the public imagination with rainforest deforestation. Hong finds that McDonald’s has actively worked to frame social and environmental challenges (like unemployment and deforestation) as external issues associated with outside suppliers, and to partner with actors like Greenpeace to bolster its legitimacy (2012: 159).

Other researchers have looked at fast-food advertising and suggested that masculine burger advertisements work to defend burger consumption against metrosexual masculinity and feminized vegetarian foods (Buerkle, 2009; Rogers, 2008). A study by Brewis and Jack (2005) looks at how nostalgia works in fast-food television advertising to appeal to the ‘good old days’ when food was simple, tasty and straightforward, pushing back against cultural impressions of fast-food meat as industrialized, highly processed and ‘unnatural’.

These studies provide important context for investigating our question of how fast-food companies are working to defend meat consumption, especially the iconic hamburger, in the face of growing public awareness of the linkages between meat eating and socio-environmental externalities. In this article, we attempt to build on prior research from a diverse array of sources to examine how market actors work as moral entrepreneurs to morally affirm their products in the face of a widespread criticism of meat-eating – and fast-food burgers more specifically.

## The Moral Economy Perspective and Moral Entrepreneurship

The creation of ‘good’ and ‘bad’ food is both an aesthetic conversation (i.e. does the food taste good? look appealing?) and a moral conversation (i.e. is it morally acceptable to eat this food?). The issue of delicious food is rarely framed as separate and apart from a general category of ‘goodness’. This is especially true in a contemporary foodscape which is dominated by discourses of healthism and obesophobia (Crawford, 2006; Guthman, 2011). According to these dominant, moralizing ways of thinking, good citizens are people who pursue the good life by taking care of their bodies and enjoying good, health promoting foods; bad citizens are those who pollute their (fat) bodies with unhealthy, bad foods, such as fast-food burgers and sugary soft drinks. Other sources also suggest that fast-food meals tend to fall at the bottom of moral hierarchies of daily foodwork practices, while the upper echelon is reserved for parents whose children love eating vegetables (e.g. MacKendrick, 2018: 139, 132).

While the food system is linked to moral debates, so is the broader economic system of capitalism. A significant tradition of sociological thought emphasizes how markets and morality are co-constituted. The moral economy perspective is rooted in classic works by E. P. Thompson (1971) and Karl Polanyi (1944), who both studied the ways that market operations (and labour exploitation) are facilitated by moral frameworks that give meaning to capitalist expansion and social dislocation. Sayer helpfully defines moral economy as ‘the study of the ways in which economic activities, in the broad sense, are influenced by moral-political norms and sentiments, and how conversely, those norms are comprised by economic forces’ (2000: 80). Classic writing by Polanyi, as well as more recent works like Sayer (2000), explore how market dynamics involve moral logics that rationalize capitalist processes, but also involve a drive to provide social and ecological protection against capitalist dynamics that prioritize profit maximization and growth.

Economic sociology scholarship includes themes and premises that overlap with and are complimentary to moral economy scholarship, insofar as it emphasizes that markets are always, and inevitably, imbued with moral logics – even when these logics slip below the surface and are naturalized, invisible to the casual observer (e.g. Zelizer, 2011). Like classic works in moral economy, economic sociologists insist that the market is not a separate entity that exists apart from moral considerations. Instead, markets are conceptualized as ‘explicitly moral projects, saturated with normativity’ and involve ‘more or less conscious efforts to categorize, normalize and naturalize behaviors and rules that are not natural in any way’ (Fourcade and Healy, 2007: 299–300). Moral logics are embedded in economic institutions, but they are not automatic, or pre-determined; they involve human agency which can be observed in everyday moral sentiments or ‘lay normativities’, which involve a habitus-level sense of right and wrong (Sayer, 2007: 264; see also Wheeler, 2018).

An important project for moral economy scholarship – and economic sociology – is to unearth and assess taken-for-granted legitimations of market relationships (Fourcade and Healy, 2007; Sayer, 2007: 264). Scholars have long talked about the moral implications of the market, but new lines of inquiry focus on understanding how boundaries of moral/immoral and legitimate/illegitimate are socially constructed in the marketplace, and how these boundaries shift alongside ‘technological change, the mobilization of interested groups, or the efforts of moral entrepreneurs’ (Fourcade and Healy, 2007: 301).

We follow up on Fourcade and Healy’s suggestion that moral boundaries in the market can be repositioned by moral entrepreneurs. The concept of moral entrepreneurs was introduced by Becker (1963) to refer to the people who advance a moral agenda through defining some behaviour as deviant and morally questionable vs. socially acceptable and legitimate. The concept of moral entrepreneurialism has been productively applied to the work of government agencies and offices (Chauncey, 1980), NGOs (Felner, 2012), and professions (Scull, 1975). Moral entrepreneurialism has also been located among diffuse individuals within a cultural space stemming from ‘the full panoply of cultural performances deployed by such individuals’ (Taylor, 2010: 303). In recent decades, the growth of ‘ethical’, ‘political’ or ‘conscientious’ consumption has facilitated the entry of corporations into explicitly moralized domains (Lewis and Potter, 2011). As ethical consumption has highlighted the moral dimension of consumption, an opportunity has arisen to explore the role of corporations as moral entrepreneurs. In this article, we apply the concept of moral entrepreneur to the corporations we study to situate our analysis of their actions as efforts to shape moral evaluations of consumer behaviours. We do so to identify and better understand efforts by market actors – marketing strategies and tools – to reclaim the burger as a good food in the face of social and environmental challenges.

## Methods and Data Analysis

We selected three case studies of the selling of hamburgers – the fast-food restaurant chains of McDonald’s, Wendy’s and A&W. Our method closely resembles the extended case method (Burawoy, 1988), which begins with findings of prior research and uses in-depth analysis of a limited number of cases in order to refine and extend existing research. Our analysis of cases relies on critical discourse analysis (Fairclough, 1992). As such, we are concerned with observing and interpreting the messages present in the cases, both linguistic and visual, remaining sensitive to the relationship of the messages to the political-economic contexts in which they appear. Critical discourse analysis prioritizes attention to the ways that discourse can operate hegemonically. In our study we are particularly concerned with how the marketing of hamburgers can maintain the status quo of high rates of hamburger consumption: how does the discourse in our cases allow hamburgers to make sense and to seem like a good consumption choice?

We selected our three case studies based on a number of criteria. First, they are three of the largest distributors of hamburgers in Canada, where our study took place. Second, the two larger chains also have an international presence beyond Canada, and therefore are relevant beyond this national context. Third, these three firms have not only conventional advertising and websites but also social media presences, all of which are key venues through which each restaurant chain’s discourse is communicated to consumers. Fourth and finally, these firms represent a range of scales in the fast-food sector, ranging from McDonald’s global hegemonic presence (37,800 outlets worldwide) to Wendy’s as a medium size restaurant chain (6,500 outlets), to A&W’s relatively small size in the market (850 outlets). To the extent that marketing campaigns might vary with firm size, our cases will allow us to observe such a difference.

We observed each restaurant’s discourse in a number of ways. First, we documented in detail messages contained in linguistic and visual texts on their websites. Second, we did the same for their social media presence, specifically their Instagram and Twitter accounts. For websites and social media content, we analysed what was available to Canadian audiences. In the case of McDonald’s, their main website contains a link to pages that are specifically about McDonald’s Canada’s operations. We examined both the main pages directed toward American and international audiences as well as pages that were specifically about Canadian operations. McDonald’s has separate social media accounts for each country with nationally specific content. We examined both US and Canadian accounts to access the variability – or lack thereof – in their messages, given the company’s Canadian-specific sustainability initiative. In the case of Wendy’s, the content of their website was not nationally specific, nor did their social media content regarding sustainability vary by country. As such, we analysed their official US website and social media account. Lastly, A&W has a Canadian-targeted website and a Canadian-specific sustainability campaign, and they also have social media content that is specific to their Canadian restaurants; we focused our analysis on this content.

Our third source of data was site visits in local restaurants to document the discourse that was presented through signage, packaging and other materials made available to consumers in the restaurants. The site visits took place in the autumn of 2019, with two visits made to each restaurant chain, with one visit in downtown Toronto and another visit in suburban Toronto. The visits produced information that was intended to triangulate the observations made of the restaurants’ websites and social media accounts.

Our discourse analysis began with sensitivity to a range of conceptual issues that had been raised by prior research. We reviewed the discourse to answer the question of how the corporations were selling the burger as a good consumption choice. How were critiques of beef managed? How was the hamburger framed as a desirable consumption choice, and did these framings serve to normalize and valorize that choice? What corporate identity was presented to the consumer to make the messages about hamburgers trustworthy and resonant?

The messages on the websites were systematically catalogued and mapped. The mapping allowed us to see both how the messages were organized for presentation to audiences and also what the repertoire of messages was, the messages’ iterations and variations, and how they collectively generated an underlying narrative. The process was undertaken as well for the messages contained in the social media accounts of each restaurant chain. The three authors together reviewed the linguistic and visual messages presented in each firm’s discourse in order to look for patterns in the messages that were relevant to answering our questions about how hamburgers were defended and rendered appealing in the face of public criticism. Through site visits in restaurants, we looked for all forms of communications with customers. We saw the same underlying narrative for each respective restaurant represented in the linguistic and visual messages present in the physical space of the restaurant, although sometimes through textual and visual messages that varied minimally from online.

## Findings

We identify three distinct discursive strategies for marketing hamburgers while managing meat critiques: (1) *global managerialism* (McDonald’s), (2) *aestheticized simplicity* (A&W); and (3) *nostalgic, personalized appeals* (Wendy’s). Our inductive analysis led us to identify and focus on both affective and informational tools. Affective tools relate to how companies evoke emotional states and produce particular moods – through images, language, and storytelling. Informational tools are the ways in which information about beef production is made available to the consumer. We discuss the proliferation and obfuscation of information, that is, how information is variously presented without restraint or to excess in some instances, or buried, veiled or clouded in other instances. These modes of information were deployed to convey a sense of response to the burger crisis, but one that did not invite critical engagement from consumers. All three companies simultaneously mobilize strategies of obfuscation and proliferation of information, be it through information dumps with little ludic content (Wendy’s); mood-setting image boards with vague textual data (A&W); extensive audiovisual content presented as a polished and authoritative inquiry (McDonald’s Canada); or through abundant but hidden information in a warren of separate websites (McDonald’s).

## McDonald’s: Global Managerialism

McDonald’s discursive strategy for morally affirming the burger is not immediately evident on their webpage. A disinterested consumer would not find messages about beef sustainability in the front page of their website, which features a minimalist design – photos of burgers against a white background, with little textual information. This is not to say that the company does not provide information about sustainability – it does, extensively so. But only for those who look for it. An online consumer interested in McDonald’s sustainability practices would have to look for the ‘Values in Action’ link at the bottom of the homepage. There, they would be taken to a menu with the options ‘Sustainability Practices’, ‘Good Food’ and ‘Good Planet’. All roads would lead them to McDonald’s corporate homepage (a separate website from McDonald’s US or McDonald’s Canada), where their sustainability messages are laid out.

By analysing the messages on McDonald’s multiple websites (US, Canada and corporate), we found that the company extols the burger through an attempt to morally affirm not only fast food but the entire business model of transnational fast-food chains. Using the slogan *Scale for Good*, McDonald’s positions itself as a global manager of myriad local sustainability projects that produce a presumed end-product of sustainable burgers. Multiple affective and informational tools are combined in order to evoke trust on the brand’s ability to achieve sustainability. We call this strategy *global managerialism.*

In terms of affective tools, McDonald’s *global managerialism* conveys a sense of ubiquity and competence that invites the consumer’s trust. Unlike Wendy’s and A&W, which emphasize feelings of simplicity, nostalgia and local production (described later), McDonald’s does not attempt to deflect attention from its position as a multinational corporation. The company proudly posits that ‘the size and reach of our business puts us in a unique position to improve people’s lives and the environment’. The *Scale for Good* campaign emphasizes McDonald’s role in ‘helping set up’ and manage ‘regional multi-stakeholder platforms’ of beef producers in Canada, the USA, Europe, and South America (McDonald’s Corporate, n.d.). By doing so, the company presents itself as a competent manager that will ‘take care’ of sustainability for their clients and suppliers, while also conveying a sense of stability and predictability – the food will remain the same. All McDonald’s asks of its consumers is trust in their management capabilities. These affective tools – global competence and stability – are conveyed through a combination of images of ‘worldly’ local producers and ecosystems (the Amazon forest, for example) and barely distinguishable images of farms around the world.

In terms of informational tools, the *Scale for Good* campaign relies on what could be called environmentalist name-dropping. The campaign draws legitimacy from its partnerships with local producers as well as transnational environmentalist organizations, which are extensively listed both in the website and on press releases. On the website, extensive texts combine expert knowledge about maintaining ecosystems intact with hyperlinks to United Nations and other international organizations’ documents and treaties. A senior member of the World Wildlife Fund is quoted praising the company: ‘as one of the largest single customers of beef globally, McDonald’s is able to engage every point along the value chain’. In a media release, the CEO of *Conservation International* states the importance of multinational corporations in the fight against climate change:

When a company like McDonald’s acts, the world can change. Food is probably the single easiest way each of us can reduce our contribution to climate change. McDonald’s is now making that a little easier with an ambitious commitment to low-carbon growth. **It’s good for our planet, good for people, and good for business.** I applaud McDonald’s for leading us toward this promising future. (McDonald’s Corporate, n.d.)

In short, McDonald’s *Scale for Good* campaign posits the company as a global entity that manages sustainability projects across the world, affectively conveying both competence and stability. In terms of informational messages, the company engages in a *proliferation* of information through extensive references to environmental organizations and regulations, United Nations’ documents, and expert knowledge when discussing local sustainability projects. However, this information is not easily available online; a consumer hoping to understand McDonald’s messages about beef sustainability needs to go through many hyperlink rabbit-holes through multiple webpages to access this information. At the same time, this strategy legitimates the company’s business model, converting something considered by many as negative (its scale as a transnational corporation) into something good for ‘people, business, and the planet’.

McDonald’s messages about global sustainability are not only concerned about the company’s position as a global entity. Local production is also important to the company’s affirmation of the burger. McDonald’s Canada’s campaign about local, sustainable beef is hailed as a jewel in the corporate crown – a successful case in the larger company’s ‘journey towards beef sustainability’ (McDonald’s Canada, n.d.). In terms of affective tools, McDonald’s employs a language of futurity and potentiality when presenting its ‘beef sustainability’ projects. Page titles such as ‘Our Sustainability Journey’ are juxtaposed against images of a male-presenting white farmer looking ahead into the open plains. The company’s sustainability efforts are metaphorically described as a ‘journey’ or a ‘road’ in which the company is moving forward (‘This isn’t the easy road, it’s the right road’). The company offers a proliferation of affective images and normative language. The text and visuals convey the sense of ‘futurity’ typical to environmentalist rhetoric about sustainability (i.e. thinking about the future), while simultaneously obscuring the efficiency of these projects, which are incomplete (‘a journey’ without destination) and vaguely beneficial (the ‘right road’).

McDonald’s use of a language of futurity and their position as global managers of local sustainability projects reaches its apex in the case of McDonald’s Canada. In a campaign advertised on both American and Canadian McDonald’s websites, we learn that in Canada, one particular burger is being produced using ‘sustainable beef’, defined as:

At McDonald’s Canada, [sustainable beef]’s a commitment. A commitment to ensuring we preserve Canada’s most valuable resources for future generations to come. To look after the land, to care for animals, and to provide the best quality food we possibly can without compromise. (McDonald’s Canada, n.d.)

This advertising campaign is composed of six videos embedded in the company’s website (McDonald’s Canada, n.d.). The videos feature interviews with Canadian farmers in which informational and affective tools are at play. Farmers discuss how to reduce their carbon footprint and maintain the ecosystem in which cattle are raised – in these cases, the ‘grasslands’:

The same pound of beef today produces fifteen per cent less greenhouse gas than it did thirty years ago, so our environmental footprint is shrinking. (Man, middle aged)

Beef sustainability is a way to produce food so that we don’t run out of the resources that we have. (Man, middle aged)

Beef production is really a by-product of what we do. What we really do is ecosystem management. The cattle are the tools that we use to manage that ecosystem. (Man, young adult)

Information about sustainability is emphasized in these quotations, but farmers also discuss the affective meanings they ascribe to sustainability. We see this primarily through a theme of futurity that explicitly invokes children and the generational transmission of land as part of the work they are doing to produce McDonald’s beef:

From a ranchers’ perspective, sustainability means that I get to take this land right here and pass onto the next generation. (Woman, middle aged)

That’s the reason we do it, for our kids and grandkids. (Man, middle aged)

My thing with sustainability is that, now having a daughter [speaker tears up; close-up of a toddler], it allows me the opportunity to make her a part of it, ‘cause if we don’t take care of it, she won’t have an option. (Woman, young adult)

Particular to McDonald’s approach to sustainability is how images of futurity are explicitly tied to economic viability, prosperity and inter-family farm succession. While Canadian ranchers interviewed for these ads emphasize the importance of environmental sustainability for children in general, they also focus on how sustainability is important to the transmission of property within families. Affective messages about family are combined with informational tools that convey expert knowledge about farming efficiency. Both messages come together under the guise of future economic prosperity: creating a prosperous business to pass along to the next generation of beef farmers.

In McDonald’s restaurant spaces, we see the online affective and informational tools reproduced in physical form, working to convey a sense of efficiency and competence that is characteristic of global managerialist messaging. The décor in the restaurants we visited was vaguely upscale and modern. It featured minimalist aesthetics, a subdued, modern colour palette, and minimal messaging about cattle, farms, burgers or sustainability. In fact, the dominant visual aspect of the interior space was a poster that covered an entire wall to promote the McCafe portion of the restaurant, which positions McDonald’s coffee and pastry products as a high-quality, relatively high-end fast-food choice. The interior spaces we observed seemed designed to create a sense of modernist aesthetic restraint and competence, quality and control. The large touch-screen ordering stations presented an intuitive interface that was highly functional, modern and visually appealing, relying on uncluttered text and a preponderance of white space. Overall, the space of the restaurant stylistically mirrored the broader narrative of competence and professionalism. With respect to meat in particular, the only information we observed was on the packaging for a hamburger we ordered, which arrived in a cardboard container with the brief message ‘not without Canadian beef farmers’. This slogan echoes the sourcing information about the beef found online that emphasizes the goodness (read: sustainability) of Canadian ranching practices. The paucity of hamburger-specific information, which is probably all that many customers will come to know about the beef McDonald’s serves, may contribute to an image of McDonald’s as a well-managed chain able to provide quality food experiences at a large scale, with reliability and integrity; consumers can enjoy a high-quality coffee, without worrying too much or thinking too hard about whether the burger they enjoy is sustainably produced.

In sum, McDonald’s – both in Canada and in the USA – combines affective and informational tools to convey that eating burgers is good for ‘people, business, and the planet’, as mentioned by the CEO of *Conservation International*. Affectively, they convey competence and trust through the *Scale for Good* slogan; and futurity and prosperity through the ‘A More Sustainable Future’ campaign. In terms of informational messages, on the one hand they provide extensive expert knowledge about sustainability – through numerous documents, regulations, and media releases about partnerships. This information, however, is not clearly accessible to a casual consumer who visits the homepage menu or goes to the restaurants – it is available only to those who actively look for it online. On the other hand, obfuscation of information occurs in the more easily accessible messages about meat production, that is, the Canadian campaign about ‘sustainable farmers’. In this section of the website, language about sustainability is vague. The specificities and the actual efficacy of their sustainability project are not discussed. This vagueness is best illustrated by the wordplay found in the restaurant’s cardboard burger container: ‘*not without* Canadian beef farmers’. This wording symbolically nods to the importance of ‘local’ beef production but does not commit McDonald’s to any particular standard of sustainability to which it could be held accountable.

## A&W: Aestheticized Simplicity

Like McDonald’s, A&W Canada’s homepage does not provide the user with sustainability messages right away. The curious consumer must follow a similar path as with McDonald’s and go through the successive hyperlinks ‘Our values’, ‘Our food’, ‘Proteins’, and lastly ‘Beef’, to find the company’s information on beef sustainability. A&W presents an advertising campaign that has some similarities to that of McDonald’s Canada, featuring Canadian ranchers.^2^ Unlike McDonald’s, however, A&W has a more aestheticized approach and less textual content, which centres on attractive green imagery and short narratives about families who raise cattle. The key theme found in the A&W strategy is *simplicity* – both in terms of its green aesthetics and information. There is no detailed information on sustainability practices, and unlike McDonald’s, they do not reference expert knowledge on how to maintain ecosystems. Instead, images of ranchers and cattle grazing on open plains are accompanied by short captions that hint at sustainability projects (e.g. grassland management). Visually, the pictures suggest a ‘less is more’ aesthetic of green minimalism which employs clean design and ample earth-tones imagery.

Eschewing long explanations or pages with abundant text, the website showcases appealing visuals of grasslands combined with minimal texts suggesting sustainable ranching practices. The slogan ‘Keeping it Simple’ appears juxtaposed to an image of two farmers (a man and a woman) on horseback, accompanied by their dog and standing in the grasslands. Underneath, smaller images showcase cattle roaming free in open plains, the same farmers riding horses among the cattle, and a close-up of the grass. Each image is followed by short captions like ‘natural process’ and ‘respecting the land’ (A&W Canada, n.d.a).

Information about cattle raising practices is mostly concentrated in a 2:55 minute video featuring interviews with ranching families, who are depicted as hard-working, straightforward rural people committed to their farms and ‘doing what’s right for the animal’. The interviews show how each rancher supports a ‘simple’ approach to beef production. Most of the farmers are depicted riding horses (rather than driving ATVs) to herd cattle; they wear cowboy hats and are shown in multiple scenes walking or riding horses with their children. A key theme of this video is how their ‘simple’ approach to beef production allows them to raise cattle without the use of a growth-promoting hormone implant. In the words of one farmer featured in the video, ‘ranching is an amazingly simple endeavour. The hard part is keeping it simple’ (A&W Canada, n.d.a).

Through its campaign slogan, ‘Keeping It Simple’, A&W reinforces a link between simplicity and ‘natural strategies’ that serve discerning consumers and the environment: ‘As stewards of the land, Ross and Christine keep it simple in order to maintain a sustainable ecosystem*’*. The details of ‘simple’ and ‘natural’ ranching practices are left vague. What *is* clearly established is a strong, affective connection between *local beef producers*, *nature* and *health.* This is accomplished through messages that emphasize the lack of hormones or steroids in the beef and ranchers’ rootedness in local grasslands where cows ‘naturally’ graze for their food – *contra* popular negative perceptions of factory farms where cows are confined in small stalls. The campaign features idyllic images of cattle raised in open plains and ranchers horseback riding with vague language about ‘nature’ (‘their modern approach to ranching is letting nature do its job’). A&W’s simple, ‘less is more’ approach communicates a win-win model of fast-food: their hamburgers are all the more delicious *because* they are good for the environment. Affectively, they convey the beauty of a simple approach that is linked to natural ways of raising cows and to better burgers for consumers.

But while the campaign focuses on local producers rooted in specific grassland contexts, and features several Canadian ranch families located in different provinces, their beef patties are not entirely produced locally, as per the text in their website:

For us, great burgers come first. So it’s only natural that we’re the first and only national burger restaurant in Canada to serve beef raised without artificial hormones or steroids, from select ranches in Canada, the US, Australia and New Zealand. We’re committed to offering Canadians burgers they can confidently enjoy, free of additives, fillers or preservatives — just 100% pure beef. (A&W Canada, n.d.a)

Here, information on transnational meat production is obfuscated by vague language around ‘Canadianness’ and beef. ‘Canadian burgers’ are not necessarily burgers made of Canadian beef, but rather a ‘100% pure beef’ burger – made in Canada or elsewhere. Locality becomes a nexus for ‘natural’, which in turn signifies the lack of additives and fillers in the meat, not the practices of raising cattle and processing the beef.

A&W Canada was the only company among those analysed that extended its message of simple, aestheticized sustainability to its social media platforms. In June of 2019, images of ranchers, farms and cattle were available on the company’s Instagram account. Like the website, their Instagram feed is highly aestheticized: it combines stylized photos of farms, most of them aerial images of open plains with cattle roaming free, with a heavy emphasis on a natural colour palate featuring various shades of green. Some of the images depict snow, in what might be an attempt to aesthetically localize these farms in Canada by using winter imagery. The images are accompanied by short and vague captions and are geographically localized (that is, the images are linked to a map)^3^ (A&W Canada, n.d.b).

In short, our analysis of A&W discourse suggests that the firm invests heavily in affective tools and in obfuscation of information to morally affirm the burger and beef consumption, producing a deeply aestheticized image of sustainability as natural, green and local while providing little information about sustainability practices. A&W’s marketing material does draw attention to its use of hormone-free cattle to make its burgers, a message that arguably appeals to health-conscious consumers seeking a hormone-free burger (rather than an environmentalist seeking specific information about sustainable beef production). Claims about ‘natural’ ranching methods and sustainable production techniques are conveyed less through specific textual claims, and more through appealing visual images that emphasize simple design, open-spaces and green landscapes where cattle can forage freely.

**Figure 1.**
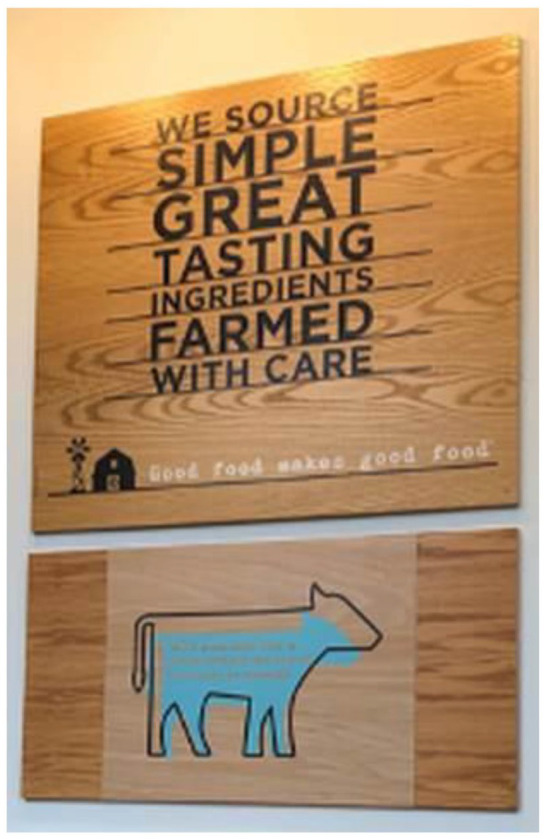
A&W Restaurant Signage.

This mix of affective and informational tools was powerfully present in the physical spaces of A&W restaurants. In the restaurants we visited, the décor was highly stylized and heavily featured green imagery of farms and fields, as well as wood/wood-like décor, as shown in Figure 1. The largest difference from McDonald’s was in the salience of informational messages, as the A&W restaurant space contained more textual information about food sourcing practices and their corporate philosophy, with a heavy emphasis on ‘simplicity’ and ‘naturalness’. Animals, farms and burgers featured prominently in abstract, simple, earth-tone images.

Such messages were present on myriad signs on nearly every horizontal surface, on the food packaging, the placemat on the tray, and the napkins. The messages covered issues of environmental sustainability (e.g. on the napkins is the message ‘Ingredients sourced with care deserve environmentally friendly napkins.’), as well as animal welfare and health. The colour scheme and claims presented on their Instagram account were replicated within the store space, which echoes themes of farming with care and avoidance of steroids and hormones in the raising of cattle. Significantly, the messaging at the same time highlights aesthetic concerns, both in references to the ‘great tasting’ food, and also through the artistic visual style. The wood material of the sign references a simple, mid-century modernist style, and the black outline of a cow superimposed on a blue cow figure is an artistic representation of the animal. There were many comparable signs throughout the restaurant. Through the marketing materials, as well as the signs and décor at A&W, the affective and informational messages came together to achieve the strategy of aestheticized and simple sustainability.

## Wendy’s: Fresh Nostalgia

In a striking contrast with A&W and McDonald’s, Wendy’s does not rely on idyllic images of ranchers and cattle running free in its marketing materials. Instead, the company relies on *nostalgia* and *freshness* as affective messages to affirm the burger. *Contra* a negative public image of conventional mass-produced, frozen burgers, Wendy’s claims that their burgers have always been ‘fresh, never frozen’. Unlike McDonald’s minimalist white spaces, or A&W’s earth-toned abstract images of farms, Wendy’s features numerous photorealist images of meaty burgers. More broadly, they highlight the positive qualities of their romantic past of cooking fresh, delicious burgers (see Brewis and Jack, 2005), and minimize the company’s engagement with future challenges like sustainability. In a post on Wendy’s blog titled ‘A History Lesson on Deliciously Different’, Wendy’s situates their burgers in a nostalgic historical moment:

Picture this: Richard Nixon takes office; The Beatles perform on the rooftop of Apple Records to an audience dressed in fringe, vests and flared pants; and in between trips to the moon by Apollo 11 and 12, ‘The Brady Bunch’ premieres on TV.

The year is 1969 . . . and Dave Thomas is searching for a real hamburger made fresh. When he couldn’t find one for a fair price, Dave embarked on the opportunity to open his own restaurant in Columbus, Ohio, naming it after his 8-year-old daughter, Wendy.

Starting Day 1 in 1969, Dave built his restaurant on the promise that ‘Quality is Our Recipe’ – beginning with fresh beef. And now, Wendy’s Fresh Beef tradition continues, never cutting corners on taste or quality. (The Square Deal, 2018a)

Wendy’s slogan, ‘Fresh Never Frozen’, accompanies every message and informational blurb about beef in their website. The word ‘freshness’ appears to be a proxy through which the company conveys associations of fresh, wholesome food that is not globally shipped, and is instead sourced ‘locally*’*. In a ‘Frequently Asked Questions About Beef’ page, this connection between fresh burgers and ‘local’ procurement (read: North America) is repeatedly made:

We keep our beef fresh by sourcing it from North American cattle ranches so close to our restaurants that we don’t have to freeze it, unlike some of our competitors who freeze their beef because they’re getting it from faraway places . . . While some others source their beef from as far away as Australia, we stick close to home, so we can ship it fresh to our restaurants. (The Square Deal, 2018b)

The link between freshness and ‘local’, nearby sourcing is also presented in nostalgic terms, pointing to the past of the brand – their burgers have always been fresh, and therefore, ‘purposefully sourced’. ‘Local’ sourcing of fresh beef is framed as a consequence of their commitment to ‘freshness’ and deliciousness:

With purpose. On purpose. Purposeful . . . It’s meant going against the grain of conventional fast food to insist on fresh beef for our hamburgers rather than frozen patties . . . Ironically, this idea of purposeful sourcing wasn’t actually on purpose. It grew out of our decades-long quest to find the best quality ingredients to make the best tasting hamburgers. (The Square Deal, 2019)

Wendy’s emphasis on ‘freshness’ is also commonly reproduced on their social media platforms. The company is known for being playful and sarcastic on Twitter, a feature which works rhetorically to position their message as personal, and individualizing, setting the company apart from negative associations of anonymous fast-food corporations. Through funny online jokes and interactions, Wendy’s may appear more like a wise-cracking friend, and less like a faceless corporation that promotes social and ecological harms through its products. Much of the humour of Wendy’s social media presence derives from their open confrontation of other fast-food chains, especially McDonald’s. On the platform, Wendy’s opposes McDonald’s frozen burgers with their own ‘fresh, never frozen’ products. One tweet from their account, posted on 6 March 2018, states: ‘Hey @McDonalds, heard the news. Happy #NationalFrozenFoodDay to you for all the frozen beef that’s sticking around in your cheeseburgers.’ Another tweet, this one a reply to a tweet on McDonald’s account, posted on 30 March 2017, reads: ‘So you’ll still use frozen beef in MOST of your burgers in ALL of your restaurants? Asking for a friend.’ For Wendy’s, ‘fresh’ seem to function as a moral counterpoint to the common association between burgers and heavily industrialized, mass-produced frozen food. By positioning its burgers as superior alternatives to McDonald’s, a brand known for its industrial and processed burgers, Wendy’s establishes a connection between freshness, flavour, and morality.

In terms of informational tools about its beef production line, Wendy’s website engages in information proliferation, to the extent that it creates an *information dump*. Of all three brands, Wendy’s is the one that provides the most readily available information. But unlike its counterparts, there is no effort in aestheticizing most of this information: website users who look for information on animal welfare and beef sourcing will be redirected to pages filled with reams of text and just a few images, mostly logos and small graphics. Consumers are not lured into these webpages with appealing aesthetics, but the loosely organized information is there to satisfy an interested party.

Wendy’s online presence also has another peculiarity: aside from their homepage and social media pages, the company runs a blog, *The Square Deal*, where press-release-like stories are posted. There is no clear sustainability category nor an obvious ‘green consumption’ tagging system – posts about sustainability and beef sourcing do exist, but are mixed in together with other posts filed under ‘food’ and ‘community’. Thinking about these marketing materials together, it appears as though Wendy’s strategy is to divert, or at least not draw attention to concerns about the sustainability of beef products. Instead, it aims to moralize the Wendy’s burger through positive associations with nostalgic imagery, freshly procured meat patties (depicted using vivid, photographic realism), and a quirky online persona that presents as a wise-cracking, friendly figure.

**Figure 2.**
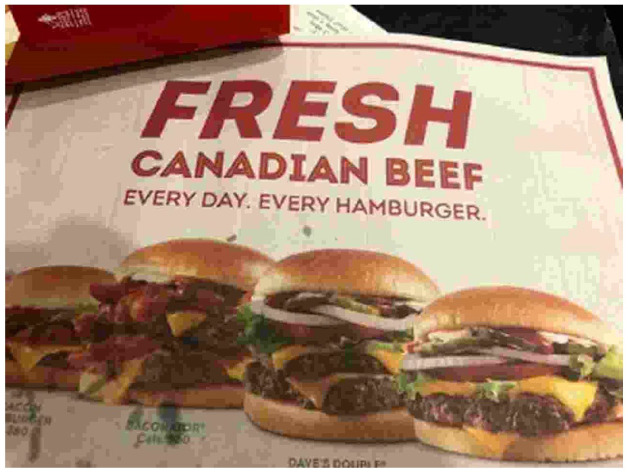
Placemat in Wendy’s.

Within the restaurant spaces, the same online affective and informational tools emphasizing nostalgia, freshness and deliciousness are apparent. One of the restaurants we visited displayed a sign near the entrance that said: ‘Welcome to real, Welcome to fresh.’ This sign’s message used plain language to communicate a down-to-earth, traditional welcome to consumers, and symbolically conveyed values of sincerity and traditional, ‘real’ food preparation methods using fresh meat – as opposed to the frozen, long-distance commodity chains associated with McDonald’s. One of the most prominent signs inside both restaurants, presented on a large banner positioned behind the cash registers, stated ‘100% Fresh Never Frozen Canadian Beef’, a repetition of one of the most prominent informational messages made online. There were quotations from the founder, Dave Thomas, in other signs, again highlighting Wendy’s ties to tradition and to a specific personality (‘Dave’) who conveys trust and quality. Figure 2 shows the placemat we received in the restaurant and its affective and informational messaging. Freshness and Canadian sourcing are the predominant aspects of the hamburgers chosen for highlighting.

Of all the brands, Wendy’s presents a large disjuncture between their affective and informational messaging. While there are scattered references to sustainability practices in their website and on scattered *Square Deal* blog posts, they do not have a coherent strategy that merges affective and informational messages about sustainability and beef. Sustainability information resides in the background of website pages, while the spotlight shines on an affective message emphasizing old-fashioned, delicious-looking burgers made with fresh meat. This focus might be due to Wendy’s claim to their ‘classic’ status: their affective messaging about ‘classic burgers’ precedes the sustainability era in marketing. Precisely because nostalgia is their main affective tool, more contemporary marketing strategies related to sustainability have little part to play in their branding and are instead conveyed through textual dumps that are not designed to draw in widespread audience, but rather tend to obfuscate. Wendy’s also appears to rely on a semiotic ambiguity regarding the meaning of ‘fresh’, ‘never frozen’, and ‘Canadian’ beef. Although these characteristics are not in practical terms related to the animal welfare, environmental, or health criticisms of beef, they may nonetheless assuage customers’ potential concerns – or at least combat negative associations of fast-food burgers as mass-produced, industrialized, and frozen. By highlighting aspects of their beef sourcing that are often associated with small-scale or local food production, and associating their brand with a specific, likeable persona (through a funny Twitter handle, or through images of founder Dave Thomas) they might be invoking for customers an image of their hamburgers as addressing, however opaquely, prevalent criticisms of beef as part of an anonymous, harmful mass-market commodity.

## Discussion and Conclusion: Moral Entrepreneurs in the Fast Foodscape

In this article we document and analyse the marketing discourses employed by three fast food chains to sell hamburgers during a time when the moral standing of the hamburger is under attack. The three distinct strategies we identify at McDonald’s, A&W, and Wendy’s all rely on combining affective and informational tools to present the hamburger as an appealing, legitimate, ‘good’ consumption option. We have shown how fast-food corporations work as moral entrepreneurs that intervene in market-culture spaces to attempt to shape moral evaluations of food choices (Fourcade and Healy, 2007: 301).

The three strategies were similar insofar as they presented hamburgers as worthy food choices and did so with both affective and informational tools. However, they were different in the extent to which they relied on one tool over the other. Moreover, the strategies contained different messages that were related to each firm’s brand identity. For McDonald’s, the goodness of a burger depends on the firm’s capacity as a transnational manager of sustainability issues. A casual McDonald’s consumer may not think about issues of meat sustainability when they walk into a restaurant, with its minimalist signage and modern aesthetic, but a worried McDonald’s consumer consulting the website can rest assured that a responsible force much larger than them is handling the issue, working to make sure that meat comes from sustainable farms around the world. In the case of A&W, its moral entrepreneurship hinges on an affective and informational strategy emphasizing a simple message of sustainability and green aesthetics. Of all three firms, the A&W strategy works the most explicitly (albeit superficially) to position their burgers as a sustainable choice that protects virtuous family farmers who act as ecological stewards. The strategy of moral entrepreneurship at Wendy’s largely sidesteps environmental critiques of meat consumption, filing this information away in plain webpages, and instead rests on its capacity to harness affective themes of nostalgia. *Contra* a negative image of frozen, industrial, assembly-line patties (that is linked to McDonald’s through its social media interventions), Wendy’s conveys a personalized image of its founder, Dave, flipping old-fashioned fresh-meat burgers sourced from Canadian farms. Among the three cases, Wendy’s presents the weakest form of moral entrepreneurship to the extent that it does not emphasize sustainability issues directly in the marketing that is most visible. Future research could explore how and why different firms adopt different forms of moral entrepreneurship.

Moral entrepreneurship is becoming a more common project among corporations as interest in and awareness of ethical consumption has increased in recent decades (Lewis and Potter, 2011). Concerning foods, beef is just one example, with others being meats such as chicken and pork, and foods that involve significant labour exploitation such as some chocolate from Africa or some Californian strawberries. We speculate that the ways in which corporations selling these foods will engage in moral entrepreneurship will correspond to the nature of the critiques being made (e.g. labour justice concerns vs. environmental concerns), as well as to the qualities of the foods (e.g. meat vs. plant).

As our study focuses on marketing content, we cannot speak to how these moral entrepreneurship projects resonate with consumers – a project that is deserving of future research. Going out for fast-food hamburgers remains very popular, raising the question of what role moral entrepreneurship plays. Eating fast food, and dining out more generally, incorporates aesthetic, social, and economic dimensions (see Warde et al., 2020). To what extent is moral entrepreneurship for the hamburger influential in consumers’ enduring willingness to consume hamburgers so frequently? Research on consumers might also illuminate the significance of the potential slippage between the target audience for the marketing vs. the consumers of the fast food – they are not necessarily the same people. This interesting potential divergence reflects the insight that marketing content must be studied not only for how it might influence consumer behaviours directly but also for its potential broader, indirect cultural effects on public discourse (Schudson, 2013).

Our data are based on Canadian case studies, and future research could explore the extent to which the tools we identify are present in cases of corporate moral entrepreneurship in other national contexts and for other kinds of firms beyond the restaurant sector. We expect that our findings have relevance beyond the national context we studied in part because the ‘grammar’ of marketing evolves within a transnational field, and also because our cases include data from McDonald’s American website, and from Wendy’s globally available Twitter account. Further research should explore potential differences in the tools of moral entrepreneurship across regions of the globe, for instance through a comparison of marketing discourse in the Global North and Global South. Moreover, case studies of different fast-food firms might also reveal different tools for moral entrepreneurship, especially within a different market sector like the ‘fast casual’ which targets a more ‘elevated’ yet convenient experience. Outside of the restaurant sector (e.g. automobiles), moral entrepreneurship could employ still other tools and strategies.

At the broadest level of analysis, as burgers increasingly become symbols of environmental disaster, corporations are called upon to deflect, reframe, and negotiate their roles in environmental practices. As suggested by Burawoy (2013) in his interpretation of Polanyi’s *great transformation*, the third and current stage of capitalism (from the 1970s to present time) is characterized by the global commodification of nature and by civil society’s resistance to the impending environmental catastrophe. We argue that moral entrepreneurialism in the context of the fast-food industry works to counter and reconcile the inherent contradictions of global capitalism – of which transnational burger chains are a powerful cultural symbol – as consumers and civil society become increasingly concerned with its ecological impacts.

Our goal here has been to document the extensive efforts made by firms to use affective and informational tools to position their foods not just as delicious, but as morally *good*, especially in a larger socio-political context where meat-eating has been challenged. Our findings highlight the extent to which corporations can veer far from the stereotypically amoral market actor trope. Instead, we see strong moral stances amongst these corporations, stances that also happen to be self-serving, but that nonetheless contribute to broad cultural ideas of what constitutes good food and responsible consumption. Although scholars have looked at corporate pressure to deliver corporate social responsibility and ethical consumption, especially in relation to alternative food, (e.g. Johnston et al., 2009), our study suggests that scholars of culture, markets and moral boundary work need to seriously consider the work that corporations do as moral entrepreneurs shaping consumer culture in mass-markets.

## Notes

1 The American-based A&W and Canadian-based A&W chains have been organizationally separate for decades and do not share marketing campaigns. Our focus here is the Canadian-based A&W operations.

2 Following the dates of their YouTube videos, A&W’s campaign was uploaded in May 2017 and McDonald’s in November 2018.

3 In August 2019, when we returned to their Instagram page, these images had been deleted.
